# Treatment for Comorbid Mental Health Disorders Among Patients Treated for Opioid Disorder: The Role of a Hub and Spoke Intervention

**DOI:** 10.1007/s10597-026-01606-9

**Published:** 2026-03-20

**Authors:** Dominic Hodgkin, Shay M. Daily, Lee Panas, Grant Ritter, Maureen T. Stewart, Sharon Reif

**Affiliations:** 1 Heller School for Social Policy and Management, Brandeis University, Waltham, Massachusetts, USA; 2 Department of Health Law, Policy and Management,, Boston University School of Public Health, Boston, Massachusetts, USA; 3 Heller School for Social Policy and Management, Brandeis University, Waltham, Massachusetts, USA

**Keywords:** Dual diagnosis, Low-barrier treatment, Psychotropic medications, Medication adherence, Bipolar disorder, Depression, Opioid use disorder, Medications for opioid use disorder

## Abstract

This study assessed whether delivery of opioid use disorder (OUD) treatment through a hub and spoke (HS) model is associated with better adherence to psychotropic medication treatment, compared to usual treatment. Washington State’s HS model required each network to include at least one mental health program, so we hypothesized that it would improve psychotropic medication adherence for people with both a mental health disorder (MHD) and an OUD. The study sample comprises adult Medicaid beneficiaries who had a diagnosis of depression or bipolar disorder in Washington during 2017–2019, who also received medication treatment for OUD. We computed rates for receipt of and adherence to relevant psychotropic medication, before and after regression-based adjustment for patient characteristics with propensity score weighting to mitigate potential selection effects. The key exposure was treatment at a HS program. Patients with bipolar disorder who were seen in HS programs were less likely than other patients to fill a mood stabilizer prescription in the 30 days post-index (predicted probability (PP) = 11.2% versus 17.8%, *p* = 0.012). However, the likelihood of achieving 80% adherence to mood stabilizers in the year post-index was higher among HS patients (21.6% versus 5.9%, *p* = 0.01). HS participation was not associated with depression treatment outcomes. HS participation does not appear to be associated with greater psychotropic medication use or adherence among patients with depression, while effects for bipolar disorder are mixed. It appears that the HS model’s focus on OUD treatment initiation did not generate much positive spillover for ongoing psychotropic medication treatment.

## Introduction

Opioid use disorder (OUD) has become increasingly prevalent in many countries, despite a recent slowdown in its spread in the US (Garnett & Miniño, 2024). One important aspect of OUD is its impact on treating comorbidities, including mental health disorders (MHD) (Pettit Bruns & Kraguljac, 2023). In particular, in the US, estimates from the 2023 National Survey on Drug Use and Health suggest that 54% of individuals with an OUD also had any mental illness, and 17% of them had a serious mental illness ((Substance Abuse and Mental Health Services Administration, 2024), author’s calculation). However, many people with both OUD and MHD receive no treatment for the MHD. During 2008–2014 in the US, among people with OUD, 47% of those with mild/moderate mental illness received no mental health treatment, while 21% of respondents with serious mental illness received no mental health treatment (Novak et al., 2019). One reason for this is that MHD expertise is often not available onsite at specialty addiction treatment programs (Pro et al., 2025), and patients in programs without it may not follow up on referrals to specialty mental health care elsewhere (Brooner et al., 2013). More generally, treatment of OUD and MHD are often siloed in separate programs, despite patient preferences (Tarn et al., 2023) and researchers’ frequent recommendations for integrated treatment approaches (National Academies of Sciences, 2025).

A systematic international review in 2020 found 8 studies that reported an association between substance misuse and poorer medication adherence among psychiatric patients (Semahegn et al., 2020), confirming earlier US findings (Lacro et al., 2002; Magura et al., 2011). Some preliminary evidence suggests that receipt of medication treatment for OUD (MOUD) improves adherence to medications for the comorbid MHD, as reported in two studies of justice-involved populations who had both OUD and MHD. First, in British Columbia, among patients with both schizophrenia and OUD, the probability of antipsychotic adherence doubled in periods that were preceded by a period of adherence to methadone maintenance therapy (Rezansoff et al., 2019). Second, in Connecticut, among patients with OUD and at least one serious MHD, receipt of MOUD (methadone, buprenorphine, or oral naltrexone) was associated with improved adherence to antipsychotic medication, and this was true for each type of MOUD (Robertson et al., 2018). One possible explanation for these findings could be that successful concurrent OUD and MHD treatment reduces one barrier to MHD treatment adherence, allowing clinician and patient to pay greater attention than previously to the MHD. In addition, the OUD treatment may itself include components that directly address mental illness, e.g., psychosocial care or integrated treatment models (Moran et al., 2019). However, the British Columbia study reported that methadone was typically delivered in pharmacy settings with witnessed oral ingestion, with few integrated supports, while the Connecticut study did not describe or analyze the psychosocial supports provided.

In recent years, experts have promoted the use of low-barrier care in settings that routinely treat patients with OUD, such as primary care and emergency departments (Jakubowski & Fox, 2020). A prominent example is the Hub and Spoke (HS) model. As originally envisioned, this model classifies some facilities as hubs, where MOUD treatment is initiated, and other programs as spokes, where patients can receive ongoing maintenance treatment once initiated (Brooklyn & Sigmon, 2017). Since its original emergence, states have modified the HS model to meet their individual needs (Snell-Rood et al., 2021). In this paper, we consider the HS model adopted in Washington state, in which MOUD-experienced providers – including primary care providers and addiction treatment programs – served as hubs, while community-based providers – such as mental health clinics, law enforcement, emergency departments, and jails – served as spokes (Reif et al., 2020; Stewart et al., 2024). In Washington, the six hubs served as experts and sources of knowledge, and MOUD could be initiated and maintained at either hubs or spokes. Prior research has assessed the impact of Washington’s HS initiative on MOUD continuity and other outcomes, with few significant findings (Reif et al., 2025a), and demonstrated that outpatient psychosocial services received early in MOUD treatment increased MOUD continuity (Reif et al., 2025a). Although these prior studies controlled for comorbid MHD, they did not examine MHD treatment as an outcome.

For OUD patients with comorbid MHD, this paper thus seeks to measure whether the Washington HS model helps improve MHD treatment entry and adherence. For OUD patients with comorbid MHD, improvements in MHD treatment entry and adherence could be expected because the Washington HS model required that each network had to include at least one mental health provider (Reif et al., 2025a). Further, three of the six HS networks are led by primary care organizations with integrated behavioral health services, and two are led by behavioral health organizations, which could have led to more attention to mental health care (Reif et al., 2020). However, an alternative hypothesis could be that the HS initiative led to such focus on OUD treatment, even at those MHD-oriented settings, that less attention was paid to mental health care (a negative spillover).

The present study seeks to determine whether receipt of OUD treatment in a HS program is associated with better adherence to MHD treatment. Our focus is on medication treatment of MHD, due to relative ease of measurement, while recognizing that psychosocial treatments are also important. The resulting information should be of interest to policymakers deciding whether to expand use of the Hub and Spoke model.

## Methods

We used an observational cross-sectional study design across multiple years, comparing subjects diagnosed with both OUD and MHD who were enrolled in a site using the HS model versus comparison subjects who were not enrolled in such treatment sites. For each subject, the period for observing outcomes was the year following their initial MOUD visit (defined below).

### Data

Washington state supplied our research team with Medicaid data from 2017 to 2019 containing outpatient, inpatient/residential, and pharmacy claims, under a data use agreement. Washington also provided a database of HS patients and their initial HS visit date, with a unique research identifier that enabled matching the patient and their initial HS visit date to Medicaid claims. The Brandeis University Institutional Review Board (IRB) and the Washington State Department of Social and Health Services IRB approved this study.

### Study Sample

Building on earlier analyses using these data (Stewart et al., 2024), we selected Medicaid beneficiaries aged 18–64 with an OUD diagnosis and receipt of MOUD treatment. We excluded children because the HS model was designed for adults, and we excluded people over 65 and those dually enrolled in Medicare because we may not have had a full picture of their health care services. For all patients, the index MOUD visit was defined as the first visit with indication of MOUD after a period of 30 days with no MOUD claims. We required 30 days of continuous Medicaid enrollment after the index MOUD visit, and used only the first episode for patients with multiple episodes. We follow previous studies (Stewart et al., 2024; Liao et al., 2023; Smart et al., 2023) in accepting the presence of a single MOUD prescription as sufficient to identify MOUD receipt.

For the present paper, we created distinct samples for the two mental health diagnoses of interest. First, a sample of patients with a diagnosis of bipolar disorder (ICD-10CM: F31 and its subcodes) in the 90 days before index MOUD visit. Second, a sample of patients who had a depression diagnosis (F32, F33 and their subcodes) in the 30 days before index MOUD visit, but no bipolar diagnosis (hereafter referred to as depression; see Table 5 for details). A longer period was used for bipolar disorder in light of its greater chronicity (Goldberg & Harrow, 2011). These and other criteria resulted in a final sample of 6,147 patients with depression but no bipolar disorder (391 were HS), and 2,262 patients with bipolar disorder (172 were HS). For some specific analyses, samples were smaller, due to exclusion of patients who could not be observed for the entire required follow-up period, as described further below.

### Measures

We used prior research to define separate sets of medications likely to be the preferred pharmacological treatment: one set for depression and another for bipolar disorder. Based on commonly used metrics, we then created several measures for medication receipt and adherence. For depression, we defined antidepressants as the relevant set of medications. For bipolar disorder, we defined the relevant medication set as mood stabilizers, which were broadly defined to include anticonvulsants, antipsychotics, lithium salts, and long-acting injectable antipsychotic medications, in accordance with National Quality Forum (NQF) measure #1880 (see below). Medications were identified using National Drug Codes.

Medication receipt was defined as filling at least one prescription from the relevant set (e.g., antidepressant, mood stabilizer) over the 30 days after index MOUD visit, and similarly, over the year after index (for those continuously enrolled for one year). These follow-up periods have been used in prior research for these medication classes (Katon et al., 1992; Li et al., 2002).

For medication adherence, the measures differed by medication class. For antidepressants, we used two measures based on the Healthcare Effectiveness Data and Information Set (HEDIS) Antidepressant Medication Management measures (National Committee for Quality Assurance, 2025), commonly used performance metrics for quality of care. First, among those with any antidepressant use, filling antidepressant prescriptions that covered at least 84 days (12 weeks), over the 114 days post-index, which is the HEDIS measure for the acute phase of antidepressant treatment. Second, whether the patient had 180 days (6 months) of antidepressant coverage in the first 231 days post index, which is the HEDIS measure for the continuation phase. For mood stabilizers, there is no HEDIS adherence measure, so we used NQF measure 1880, which requires a Proportion of Days Covered (PDC) of at least 0.8 for mood stabilizer medications during the year post-index, among adult patients with at least two mood stabilizer prescriptions during that period (Substance Abuse and Mental Health Services Administration, 2016). For each measure, the sample was restricted to those continuously enrolled for the period in question (e.g., 114 days if the measure is based on 114 days of follow-up, 180 days if the measure is based on 180 days, and so on).

In addition, patient characteristics were obtained or computed from the Medicaid enrollment data for use as covariates in analyses. These included: age group (18–29, 30–39, 40–49, 50–64), sex (male/female), combined race and ethnicity (non-Hispanic White, non-Hispanic Black, non-Hispanic American Indian, Hispanic, and other/unknown), urban/rural residence based on patient ZIP code using Rural Urban Commuting Area Codes (U.S. Department of Agriculture; Economic Research Service, 2025), and neighborhood social deprivation index (SDI) score (0 to 100, higher scores indicate more deprivation), which we recoded into quartiles (Butler et al., 2013). In addition, we constructed the Charlson comorbidity index (0, 1, 2 or more physical conditions) from each patient’s service use in the three months prior to the initial HS visit (Quan et al., 2011). We also created indicators for any non-opioid substance use disorder (SUD) diagnosis and for receipt of any psychosocial treatment, both in the 90 days before the index visit. Given the relatively small sample sizes (both overall and for certain subgroups), we simplified several of these variables for the multivariable analyses. Specifically, we combined the various race and ethnicity groups into Non-Hispanic White (around 75% of each sample) and all other; the Charlson index into zero and nonzero; and the SDI score into lowest quartile versus all others combined.

### Statistical Approach

Patients were not randomly assigned to HS, and initial descriptives indicated differences in observable characteristics between the HS and comparison groups (Table 1). We therefore applied a propensity score weighting approach to achieve greater comparability between HS and comparison subjects on selected observable characteristics. The propensity scores for this weighting were derived from multivariable logistic regressions to predict group membership as a function of age category, gender, race and ethnicity, urbanicity, comorbidity score (Charlson), comorbid SUD, and area SDI score. Weights for comparison subjects were calculated to provide average treatment effects on the treated. Applying propensity score weights, the resulting covariate balance between groups appeared acceptable, with all standardized differences at a negligible level of between − 0.05 and 0.05 (Appendix Table 6).

**Table 1:** Characteristics of study sample (unweighted)

	Depression sample			Bipolar disorder sample		
	Total	HS	Comp	Total	HS	Comp
Sample n*	6,147	391	5,756	2,262	172	2,090
	**% of patients**			**% of patients**		
**Gender**						
Female	55.3	54.7	55.4	61.7	66.9	61.2
**Age Category**						
18–29	33.5	36.8	33.2	30.7	33.7	30.4
30–39	32.7	32.2	32.8	32.3	33.1	32.2
40–49	18.3	17.9	18.4	21.8	19.2	22.1
50–64	15.5	13.0	15.7	15.2	14.0	15.3
Race and Ethnicity**						
Non-Hispanic White	76.0	**69.8**	**76.4**	78.2	76.2	78.4
Non-Hispanic Black	4.3	**7.4**	**4.1**	4.4	7.6	4.1
American Indian	6.2	**5.4**	**6.3**	6.1	< 8% ‡‡	6.2
Hispanic	8.4	**8.4**	**8.4**	6.6	< 8% ‡‡	6.8
Other/Unknown	5.2	**9.0**	**4.9**	4.7	7.0	4.6
Urbanicity***						
Urban	86.9	85.4	87.0	89.9	91.9	89.7
**Charlson Comorbidity Index**†						
0	71.6	70.6	71.6	66.6	62.2	67.0
1	18.0	18.9	18.0	20.1	21.5	20.0
2+	10.4	10.5	10.4	13.3	16.3	13.0
**SUD Comorbidity (non-opioid)** §						
Yes	61.1	63.4	60.9	68.2	73.3	67.8
**Social Deprivation Index**						
Quarter 1	25.5	**22.0**	**25.7**	24.9	26.7	24.7
Quarter 2	24.6	**30.2**	**24.3**	24.9	26.7	24.7
Quarter 3	25.2	**21.7**	**25.4**	26.3	28.5	26.1
Quarter 4	24.7	**26.1**	**24.6**	24.0	18.0	24.5
**Prior Mental Health Medication Treatment**‡						
Yes	16.2	13.3	16.4	34.9	29.1	35.4
**Prior Psychosocial Treatment** §						
Yes	63.6	**70.6**	**63.2**	68.5	73.8	68.0

**SOURCE** Authors’ analysis of Washington State Health Care Authority Medicaid Claims

Bolded values indicate a significant chi-square difference at < 0.05; * Sample n are frequencies, all other values are percentages; ** All race categories represent non-Hispanic groups except for Hispanic. *** Urban/rural categories are based on USDA Rural Urban Continuum Area codes; † Charlson comorbidity index, where higher scores indicate greater levels of comorbidity; ‡ Prior mental health treatment includes antidepressant medications for depression sample and mood stabilizers for bipolar disorder sample; § during the 90 days before index visit. HS = hub and spoke; Comp= comparison group; SUD= substance use disorder. ‡‡ Exact number withheld to comply with HCA cell suppression policy

We estimated multivariable regression models that included the explanatory variables listed above and used the propensity score weights. There was one model for each of the seven study outcomes described above: (1) any antidepressant use in first 30 days post-index, (2) any antidepressant use in first 365 days post-index, (3) acute phase antidepressant adherence, (4) continuation phase antidepressant adherence, (5) any mood stabilizer use in first 30 days post-index, (6) any mood stabilizer use in first 365 days post-index, (7) 80% mood stabilizer coverage over the year post-index. Logistic regression was used in all models, since the outcomes were all binary. P-values less than 0.05 were deemed to indicate statistical significance. The regression results are reported as predicted probabilities (of a given outcome; PP) for different levels of each explanatory variable. In the final models, we checked for collinearity among our explanatory variables and found that no variable had a variance inflation factor higher than 1.4, which is well below a typical cutoff of 2.5 (Allison, 2012). As a sensitivity analysis, we also reestimated the models using a narrower definition of mood stabilizer (anticonvulsant class only), since some research on bipolar medication treatment uses that definition.

## Results

### Sample Description

Our sample selection criteria resulted in 6,147 subjects with depression without co-occurring bipolar disorder and 2,262 subjects with bipolar disorder (Table 1). Women comprised 55% (unweighted *n* = 3,402) of the depression sample and 62% (*n* = 1,395) of the bipolar disorder sample. In both samples, more than 60% of patients were under age 40 (*n* = 4,068 for depression, 1,424 for bipolar disorder), more than 75% were non-Hispanic White (*n* = 4,671 for depression, 1,769 for bipolar disorder), and only 10 to 13% lived in rural areas (805 for depression, 229 for bipolar disorder). In the depression sample, 16% of subjects (*n* = 998) had filled an antidepressant prescription in the 30 days before index visit. In the bipolar disorder sample, 35% (*n* = 789) had filled a mood stabilizer prescription in the 90 days before index visit.

In each sample, only a small minority of subjects were seen at HS programs: 6% (*n* = 391) in the depression sample and 8% (*n* = 172) in the bipolar disorder sample. In the depression sample, before propensity score weighting, HS patients were less likely than comparison patients to be non-Hispanic White (70% versus 76%, *p*<0.0005), and more likely to have received prior psychosocial treatment (71% versus 63%, *p* = 0.004). In the bipolar disorder sample, no patient characteristic differed significantly between study groups. Application of propensity score weighting further reduced differences between the study groups.

### Unadjusted Outcome Measures

In the depression sample, 12% of patients (*n* = 781) filled an antidepressant prescription in the first 30 days post-index, and 35% (*n* = 1,619) did so in the first year post-index (among those enrolled for that period; Table 2). Conditional on starting antidepressant treatment, 22% of patients (*n* = 827) had 84 days of antidepressant coverage in the first 114 days post index, and 13% (*n* = 428) had 180 days of antidepressant coverage in the first 231 days post index. Although each rate appeared slightly higher for the comparison group, none of these rate differences between study groups were statistically significant (all p-values > 0.06).

**Table 2:** Rates of psychotropic medication use by study group

		% of patients		
	Sample *n*	Total	HS	Comp
Depression sample				
Any antidepressant fills in the 30 days post-index	6,147	11.1	10.5	11.7
Any antidepressant fills in the 365 days post-index ^a^	4,398	34.0	32.6	35.4
84 days of antidepressant coverage in first 114 days post index ^a, b^	3,251	21.9	19.0	24.8
180 days of antidepressant coverage in first 231 days post index ^a, b^	2,800	12.9	11.1	14.6
**Bipolar sample**				
Any mood stabilizer fills in the 30 days post-index ^a^	2,262	13.7	**10.5**	**16.9**
Any mood stabilizer in the 365 days post-index ^a^	1,653	43.7	41.4	45.7
80% mood stabilizer coverage in the first year post-index ^a, c, d^	487	12.7	**20.7**	**6.2**

**SOURCE** Authors’ analysis of Washington State Health Care Authority Medicaid Claims

Bolded values indicate a significant chi-square difference at < 0.05; a= Limited to patients enrolled for the period in question. b= Limited to patients who had started an antidepressant and were observable for the relevant follow-up period. c= Limited to patients who were observable for the year post-index and had filled at least 2 mood stabilizer prescriptions. HS = hub and spoke; Comp= comparison group. Analyses used propensity score weighting (See Methods)

In the bipolar disorder sample, 14% of subjects (*n* = 404) filled a mood stabilizer prescription in the first 30 days post-index, and 44% (*n* = 782) did so in the first year post-index (among those enrolled for that period). Among patients who had at least 2 mood stabilizer prescriptions in the year post-index, 13% (*n* = 36) were covered by mood stabilizer prescriptions (adherent) for 80% of that period. The mood stabilizer fill rate in the 30 days post-index was lower (*p* = 0.029) in the Hub and Spoke group (10.5%, *n* = 18) than in the comparison group (16.9%, *n* = 386), but the adherence rate was higher (*p* = 0.004) among HS (20.7%) than comparisons (6.2%).

### Multivariable Analysis

Multivariable analysis confirmed the pattern of the descriptive results (Tables 3 and 4), with HS participation only proving statistically significant in two of the models, both for outcomes in the bipolar disorder sample. As in the descriptive analysis, a mood stabilizer prescription in the first 30 days post-index was less likely among Hub and Spoke patients than comparison group (predicted probability [PP] = 10.4% versus 17.0%, *p* = 0.009). However, the likelihood of achieving 80% adherence to mood stabilizers in the year post-index was higher among HS patients (21.6% versus 5.9%, *p* = 0.01). HS participation was not predictive of the other bipolar disorder outcome (*p* = 0.39) .

**Table 3:** Correlates of medication use: Logistic regression analysis of the depression sample

Sample size	Any AD in 30 days post-index		Any AD in 365 days post-index		84 days of AD coverage in first 114 days post-index		180 days of AD coverage in first 231 days post-index	
	*n* = 6,147		*n* = 4,398		*n* = 3,251		*n* = 2,800	
	PP	SE	PP	SE	PP	SE	PP	SE
**Study Group**								
Hub and Spoke	10.4%	1.4%	32.3%	2.7%	19.2%	2.7%	11.5%	2.5%
Comparison	11.8%	0.4%	35.6%	0.8%	24.6%	0.8%	14.2%	0.7%
**Age Category**								
18–29	10.2%	1.1%	29.5%	2.4%	17.1%	2.4%	7.5%	1.6%
30–39	11.9%	1.3%	**36.6%**	**2.4%**	22.6%	2.4%	**13.5%**	**2.2%**
40–49	13.2%	1.9%	**37.6%**	**3.1%**	24.1%	3.0%	**17.2%**	**3.0%**
50–64	8.2%	1.6%	35.3%	3.7%	**27.8%**	**4.3%**	**17.1%**	**3.5%**
**Gender**								
Female	10.34%	0.9%	33.6%	1.7%	23.6%	1.8%	13.9%	1.6%
Male	12.0%	1.2%	34.5%	2.2%	19.5%	2.1%	11.6%	1.8%
**Race and Ethnicity**								
Non-Hispanic White	10.6%	0.8%	35.2%	1.7%	25.0%	1.8%	15.0%	1.6%
Other	12.3%	1.4%	31.3%	2.5%	**14.7%**	**2.0%**	**8.1%**	**1.7%**
**Urbanicity**								
Rural	11.6%	1.6%	32.9%	3.8%	22.4%	3.6%	7.0%	1.3%
Urban	11.0%	0.8%	34.2%	1.5%	21.9%	1.5%	**14.0%**	**1.4%**
**Charlson Comorbidity Index** ^**a**^								
0	12.0%	0.9%	35.4%	1.7%	20.5%	1.6%	11.6%	1.3%
1 or more	8.9%	1.4%	30.9%	2.5%	25.2%	2.9%	15.8%	2.6%
**Comorbid SUD (non-opioid)** ^**a**^								
No	12.4%	1.3%	36.0%	2.2%	28.3%	2.7%	14.9%	2.0%
Yes	10.3%	0.9%	32.9%	1.8%	**18.2%**	**1.6%**	11.7%	1.5%
**Social Deprivation Index**								
Q1	11.1%	0.8%	33.8%	1.6%	22.1%	1.6%	12.6%	1.4%
Q2 or higher	10.9%	1.5%	34.8%	2.7%	21.4%	2.5%	13.9%	2.3%
**Prior AD Use** ^**b**^								
No	5.9%	0.6%	28.7%	1.5%	20.5%	1.5%	12.0%	1.3%
Yes	**44.5%**	**3.4%**	**69.5%**	**3.8%**	**27.7%**	**3.6%**	16.7%	3.2%
**Prior psychosocial treatment** ^**a**^								
No	10.2%	1.1%	29.3%	2.3%	22.9%	2.5%	15.6%	2.6%
Yes	11.5%	0.9%	**36.1%**	**1.6%**	21.5%	1.7%	11.8%	1.3%

**SOURCE** Authors’ analysis of Washington State Health Care Authority Medicaid Claims

Bolded values indicate a significant difference at < 0.05; (a) During the 90 days before index visit; (b) During the 30 days before index visit. AD = antidepressant; PP = predicted probability; SE = standard error. SUD=substance use disorder. Analyses used propensity score weighting (See Methods)

**Table 4:** Correlates of medication use: Logistic regression analysis of the bipolar disorder sample

Sample size	Any MS in the first 30 days post-index		Any MS in the first 365 days post-index		80% MS coverage in first year post-index	
	*n* = 2,262		*n* = 1,653		*n* = 487	
	PP	SE	PP	SE	PP	SE
**Study Group**						
Hub and Spoke	10.4%	2.0%	41.7%	4.3%	21.6%	7.3%
Comparison	**17.0%**	**0.8%**	45.5%	1.3%	**5.9%**	**1.3%**
**Age Category**						
18–29	12.3%	1.6%	41.3%	3.8%	15.4%	6.3%
30–39	16.9%	2.1%	45.0%	3.5%	16.4%	5.8%
40–49	11.6%	2.4%	48.4%	4.3%	7.4%	6.2%
50–64	12.2%	3.3%	41.1%	5.9%	7.0%	6.1%
**Gender**						
Female	13.4%	1.3%	42.5%	2.4%	15.9%	4.1%
Male	14.2%	2.0%	46.2%	4.0%	6.5%	4.6%
**Race and Ethnicity**						
Non-Hispanic White	13.5%	1.3%	45.6%	2.4%	14.5%	3.6%
Other	14.3%	2.2%	37.8%	3.7%	4.5%	2.6%
**Urbanicity**						
Rural	16.2%	4.7%	42.1%	8.8%	11.9%	6.5%
Urban	13.5%	1.1%	43.9%	2.2%	12.8%	3.5%
**Charlson Comorbidity Index** ^**a**^						
0	13.2%	1.3%	45.0%	2.7%	10.3%	3.3%
1 or more	14.5%	1.9%	41.7%	3.4%	19.5%	11.0%
**Comorbid SUD (non-opioid)** ^**a**^						
No	14.7%	2.4%	52.9%	4.1%	15.2%	5.0%
Yes	13.4%	1.2%	**40.4%**	**2.4%**	11.5%	3.6%
**Social Deprivation Index**						
Q1	13.7%	1.3%	41.3%	2.5%	10.3%	4.1%
Q2 or higher	13.7%	2.0%	50.0%	4.0%	15.6%	4.5%
**Prior MS Use** ^**a**^						
No	5.4%	0.8%	34.0%	2.5%	13.6%	4.5%
Yes	**33.7%**	**3.2%**	**67.2%**	**3.9%**	11.9%	3.8%
**Prior psychosocial treatment** ^**a**^						
No	13.0%	2.2%	43.1%	4.2%	8.1%	5.4%
Yes	14.0%	1.3%	43.9%	2.3%	13.9%	3.4%

**SOURCE** Authors’ analysis of Washington State Health Care Authority Medicaid Claims

Bolded values indicate a significant difference at < 0.05; (a) During the 90 days before index visit; (b) During the 30 days before index visit. MS= mood stabilizer; PP = predicted probability; SE = standard error. SUD=substance use disorder. Analyses used propensity score weighting (See Methods)

HS participation was not predictive of any of the four outcomes for depression (all p-values > 0.08). In the models to predict antidepressant utilization, the most consistent predictor was prior antidepressant medication use, which was associated with higher probability of filling an antidepressant in the 30 days post-index (PP = 45% versus 6%, *p*<0.0005) and in the year post-index (PP = 70% versus 29%, *p*<0.0005). In addition, conditional on starting an antidepressant, prior antidepressant medication use was associated with adherence in the acute phase (first 114 days) but not the continuation phase (first 231 days). Prior psychosocial treatment was associated with higher probability of filling an antidepressant in the year post-index (PP = 36% versus 29%, *p* = 0.019). In the models to predict antidepressant adherence, over both timeframes adherence was more likely among patients who were of non-Hispanic White race (compared to all other), and among patients aged 50–64 (compared to 18–29). In addition, continuation-phase antidepressant adherence was more likely among patients aged 30–39 and 40–49 (compared to 18–29); and among urban residents (compared to rural ones).

In the two models to predict mood stabilizer utilization, a strong predictor was prior use of a mood stabilizer, with the PP being 28 percentage points higher for prior users in the model for the 30-day period (33.7% versus 5.4%), and 33 percentage points higher for the 1-year period (67.2% versus 34.0%) (*p*<0.0005 in each case). In addition, patients with comorbid non-opioid SUD were less likely to fill a mood stabilizer in the first year post-index (PP = 40.4% compared to 52.9%, *p* = 0.008). In the model to predict mood stabilizer adherence, no explanatory variables other than HS achieved statistical significance (all p-values > 0.06), possibly because the sample was much smaller (487 instead of 2,262).

Qualitatively similar results were obtained from our sensitivity analysis that used a narrower definition of mood stabilizer (anticonvulsant class only).

## Discussion

For all four of the outcomes examined in the depression sample, our study found no association between Hub and Spoke participation and psychotropic medication use or adherence. As a result, for depression treatment, our findings do not support the hypothesis that the Hub and Spoke program would generate positive spillovers for mental health care. For the sample of patients with bipolar disorder, we found mixed associations with Hub and Spoke participation. On the one hand, patients seen in HS programs were less likely than other patients to fill a mood stabilizer prescription in the 30 days post-index, despite initiation of MOUD. On the other hand, among patients with bipolar disorder who received at least 2 mood stabilizer prescriptions in the year post-index, those seen in HS programs had better medication adherence. Overall, our findings do not suggest a strong positive spillover from the HS program for the medication treatment of these two mental health conditions. Although the initiative encouraged treatment programs to initiate patients on MOUD, some programs may not have been screening for mental health comorbidities and may not have been well prepared to provide initial comprehensive care for them. The heavy focus on MOUD initiation may thus have diverted programs’ attention from addressing patients’ MHD issues. One possible explanation could be to note that the Hub and Spoke initiative could only have generated positive spillovers to mental health treatment if it first had a sustained impact on OUD treatment. On that point, despite the Hub and Spoke initiative’s success in increasing MOUD treatment initiation (Stewart et al., 2024), it was not associated with increases in six-month continuity of MOUD treatment (Reif et al., 2025b). A previous paper suggested that treatment programs were more focused on treatment entry than on retention, in part due to the way provider incentives were designed (Reif et al., 2025b). On the other hand, we did find a positive effect of HS on bipolar medication adherence.

Our findings for antidepressant treatment differ from two prior studies which found that receipt of medication treatment for OUD (MOUD) improves adherence to medications for the comorbid MHD in justice-involved populations who had both OUD and MHD. The first of these studies concerned schizophrenia patients and was conducted in British Columbia (Rezansoff et al., 2019); the second concerned patients with multiple serious MHDs and was conducted in Connecticut (Robertson et al., 2018). One likely reason for the difference in findings is that those studies compared mental health care between populations receiving versus not receiving MOUD treatment. By contrast, in our study all subjects received MOUD treatment, and instead the comparison was between different contexts of that treatment: a Hub and Spoke model versus treatment as usual. In addition, both those studies included only individuals with criminal justice involvement, whereas our study did not impose that limitation.

More generally, MHD treatment rates for this population appear lower than some other populations reported in the literature. For example, the antidepressant adherence rates of 23% (acute phase) and 14% (continuation phase) reported here are well below the equivalent rates for Medicaid health plans nationwide in 2023, which were 62.6% for the acute phase and 45.3% for continuation phase (National Committee for Quality Assurance, 2025). One reason for our lower adherence rates might be that the national HEDIS rates were for a broader Medicaid population, not limited to individuals who were receiving MOUD treatment (as in our sample). In the case of bipolar disorder, one study of Medicaid enrollees with bipolar disorder in California found that 42.4% of patients used a mood stabilizer during the first year of bipolar disorder treatment (Li et al., 2002), which is similar to the 45% observed in our sample – except that our study used a broader definition of mood stabilizer medications. For mood stabilizer adherence, a study of patients hospitalized for schizophrenia or bipolar disorder found that during the 6-month period following a hospitalization for bipolar disorder, the mean medication possession ratio (MPR) for the second-generation antipsychotic agent prescribed at discharge was 37.3%. That study did not report the proportion of patients who achieved an MPR of 80%, although it presumably was very low (Berger et al., 2012). Unlike our study, neither of these studies was limited to individuals receiving MOUD.

Regarding covariates, our finding that adherence to antidepressant medications is lower among non-White patients confirms some earlier studies (Fleck et al., 2005; García et al., 2016). Those studies noted issues such as therapeutic alliance and patients’ beliefs about medication that were not measurable for the current study. Similarly, younger patients were less likely to adhere to antidepressant treatment (continuation measure), confirming earlier studies with similar findings (Jawad et al., 2018; Kessing et al., 2007). However, most of the other covariates we included were not systematically associated with more than one of the study outcomes.

Several limitations to our study should be noted. First, cross-sectional designs pose potential threats of selection bias, although we tried to address this by the use of propensity score weighting. In particular, claims data lack information on some key influences on medication adherence (e.g. housing status or SUD severity) which may be correlated with having a mental health disorder. For the present study, this would mainly be a bias concern if such factors were correlated with HS participation. Second, patients could have stopped adhering to MOUD after the index date, which would likely have an impact on their use of other medications. Third, the sample sizes likely prevented detection of small effects, particularly for analyses of medication adherence. Fourth, medication fills are an imperfect measure of medication adherence, as some patients may have filled a prescription without subsequently taking the medication. Finally, these results are specific to Washington state’s HS implementation, and may not generalize to other states to the extent that the programs or populations elsewhere differed from Washington’s.

## Conclusion

Our study found only limited evidence that Hub and Spoke participation was associated with greater psychotropic medication use or adherence among patients with depression or bipolar disorder, over periods from 30 days to one year after MOUD treatment initiation. This may be because the Hub and Spoke model we studied was focused on OUD treatment initiation, and this was not enough to generate positive spillovers for ongoing MHD treatment. Program designers may want to consider how to improve management of mental health issues in future refinements of the Hub and Spoke model.

## Data Availability

Data for this study was accessed through a data use agreement with the Washington State Health Care Authority. The agreement does not permit the authors to share the underlying data, but interested researchers can contact the Authority to seek their own data use agreement.

## Appendix

Tables 5 and 6

**Table 5:** Sequence of Sample Construction Criteria

	Depression		Bipolar	
Hub and Spoke (Intervention Group)	*N*	Exclude	*N*	Exclude
Any Medicaid eligibility in study period	5,792		5,792	
Limit to: Non-dual eligible (Medicare and Medicaid), CHP State only and/or family planning enrollees	5,622	170	5,622	170
Limit to: Medicaid Enrollees with indication of OUD	5,526	96	5,526	96
Limit to: Indication of MOUD 2d prior to 5d post start date	4,246	1,280	4,246	1,280
Limit to: Has depression dx in 90 days pre index MOUD	807	3,439	4,246	***
Limit to: Has bipolar diagnosis in 90 days pre index MOUD	807	***	278	3,968
Exclude if: Has bipolar dx in 90 days pre index MOUD	705	102	278	***
Limit to: Enrollee 18–64 years old at start of episode	704	***	278	0
Limit to: Enrollee has 90 days pre index and 30 days post index continuous enrollment	620	84	252	26
Limit to: Has 30-day MOUD clean period before index	391	229	172	80
Exclude if: Episode prior to August 1, 2019	391	0	172	0
**Comparison Group**	**N**	**Exclude**	**N**	**Exclude**
Any Medicaid eligibility in study period	164,514		164,514	
Limit to: Non-dual eligible (Medicare and Medicaid), CHP State only and/or family planning enrollees	154,467	10,047	154,467	10,047
Limit to: Medicaid Enrollees with indication of OUD	102,689	51,778	102,689	51,778
Limit to: Indication of MOUD 2d prior to 5d post start date	42,820	59,869	42,820	59,869
Limit to: Has depression dx in 90 days pre index MOUD	8,169	34,651	42,820	***
Limit to: Has bipolar diagnosis in 90 days pre index MOUD	8,169	***	2,525	40,295
Exclude if: Has bipolar dx in 90 days pre index MOUD	7,254	915	2,525	***
Limit to: Enrollee 18–64 years old at start of episode	7,226	28	2,515	< 12 ‡‡
Limit to: Enrollee has 90 days pre index and 30 days post index continuous enrollment	6,215	1,011	2,250	> 260 ‡‡
Exclude if: Episode prior to August 1, 2019	5,756	459	2,090	160

SOURCE Authors’ analysis of Washington State Health Care Authority Medicaid Claims

*** denotes that this criterion was not applied to this group. NOTE: ‡‡ denotes exact number withheld to comply with HCA cell suppression policy. MOUD=medication treatment for opioid use disorder

**Table 6:** Covariate balance before and after propensity score weighting

	Depression sample			Bipolar disorder sample		
	Standardized mean difference (SMD)			Standardized mean difference (SMD)		
**Covariate**	**Unweighted**	**Weighted**	**Δ**	**Unweighted**	**Weighted**	**Δ**
SUD comorbidity (pre-index)	-0.052	0.008	-0.043	-0.116	0.020	-0.096
age18_29	-0.076	0.011	-0.065	-0.064	0.000	-0.064
age30_39	0.011	-0.004	-0.007	-0.028	-0.003	-0.025
age40_49	0.011	-0.014	0.003	0.068	-0.007	-0.061
age50_64	0.073	0.005	-0.067	0.040	0.012	-0.028
Charlson index > 0	-0.023	0.014	-0.009	-0.109	0.004	-0.105
Female sex	0.013	-0.003	-0.011	-0.110	0.032	-0.078
Nonwhite race	-0.154	0.002	-0.152	-0.051	-0.006	-0.045
Social deprivation index	-0.013	-0.001	-0.013	0.093	0.008	-0.085
Urban	0.047	0.016	-0.030	-0.074	-0.007	-0.067
Prior AD use (pre-index)	0.085	-0.004	-0.081	-	-	-
Prior MS use (pre-index)	-	-	-	0.140	-0.015	-0.126
Mean SMD	-0.007	0.003	-0.004	-0.035	0.005	-0.030

## References

[CR1] Allison, P. (2012). When can you safely ignore multicollinearity? Retrieved 7/14 from https://statisticalhorizons.com/multicollinearity/

[CR2] Berger, A., Edelsberg, J., Sanders, K. N., Alvir, J. M., Mychaskiw, M. A., & Oster, G. (2012). Medication adherence and utilization in patients with schizophrenia or bipolar disorder receiving aripiprazole, quetiapine, or ziprasidone at hospital discharge: a retrospective cohort study. *Bmc Psychiatry*, *12*, 99. 10.1186/1471-244x-12-99 22856540 10.1186/1471-244X-12-99 PMC3480886

[CR7] Brooklyn, J. R., & Sigmon, S. C. (2017). Jul/Aug). Vermont Hub-and-Spoke Model of Care for Opioid Use Disorder: Development, Implementation, and Impact. *Journal Of Addiction Medicine*, *11*(4), 286–292. ttps://doi.org/10.1097/adm.0000000000000310 28379862 10.1097/ADM.0000000000000310 PMC5537005

[CR8] Brooner, R. K., Kidorf, M. S., King, V. L., Peirce, J., Neufeld, K., Stoller, K., & Kolodner, K. (2013). Nov). Managing psychiatric comorbidity within versus outside of methadone treatment settings: a randomized and controlled evaluation. *Addiction*, *108*(11), 1942–1951. ttps://doi.org/10.1111/add.12269 23734943 10.1111/add.12269 PMC3833440

[CR9] Butler, D. C., Petterson, S., Phillips, R. L., & Bazemore, A. W. (2013). Measures of social deprivation that predict health care access and need within a rational area of primary care service delivery. *Health Services Research*, *48*(2 Pt 1), 539–559. 10.1111/j.1475-6773.2012.01449.x 10.1111/j.1475-6773.2012.01449.x PMC3626349 22816561

[CR10] Fleck, D. E., Keck, P. E. Jr., Corey, K. B., & Strakowski, S. M. (2005). Factors associated with medication adherence in African American and white patients with bipolar disorder. *Journal Of Clinical Psychiatry*, *66*(5), 646–652. 10.4088/jcp.v66n0517 15889954 10.4088/jcp.v66n0517

[CR11] García, S., Martínez-Cengotitabengoa, M., López-Zurbano, S., Zorrilla, I., López, P., Vieta, E., & González-Pinto, A. (2016). Aug). Adherence to Antipsychotic Medication in Bipolar Disorder and Schizophrenic Patients: A Systematic Review. *Journal Of Clinical Psychopharmacology*, *36*(4), 355–371. ttps://doi.org/10.1097/jcp.0000000000000523 27307187 10.1097/JCP.0000000000000523 PMC4932152

[CR12] Garnett, M. F., & Miniño, A. M. (2024). Drug overdose deaths in the United States, 2003–2023. In. NCHS Data Brief. Vol 522. Hyattsville, MD: National Center for Health Statistics. https://www.cdc.gov/nchs/products/databriefs/db549.htm

[CR13] Goldberg, J. F., & Harrow, M. (2011). A 15-year prospective follow-up of bipolar affective disorders: comparisons with unipolar nonpsychotic depression. *Bipolar Disorders*, *13*(2), 155–163. 10.1111/j.1399-5618.2011.00903.x 21443569 10.1111/j.1399-5618.2011.00903.x

[CR14] Jakubowski, A., & Fox, A. (2020). Mar/Apr). Defining Low-threshold Buprenorphine Treatment. *Journal Of Addiction Medicine*, *14*(2), 95–98. ttps://doi.org/10.1097/adm.0000000000000555 31567596 10.1097/ADM.0000000000000555 PMC7075734

[CR15] Jawad, I., Watson, S., Haddad, P. M., Talbot, P. S., & McAllister-Williams, R. H. (2018). Dec). Medication nonadherence in bipolar disorder: a narrative review. *Ther Adv Psychopharmacol*, *8*(12), 349–363. ttps://doi.org/10.1177/2045125318804364 30524703 10.1177/2045125318804364 PMC6278745

[CR16] Katon, W., von Korff, M., Lin, E., Bush, T., & Ormel, J. (1992). Adequacy and duration of antidepressant treatment in primary care. *Medical Care*, *30*(1), 67–76. 10.1097/00005650-199201000-00007 1729588 10.1097/00005650-199201000-00007

[CR17] Kessing, L. V., Søndergård, L., Kvist, K., & Andersen, P. K. (2007). Adherence to lithium in naturalistic settings: results from a nationwide pharmacoepidemiological study. *Bipolar Disorders*, *9*(7), 730–736. 10.1111/j.1399-5618.2007.00405.x 17988363 10.1111/j.1399-5618.2007.00405.x

[CR18] Lacro, J. P., Dunn, L. B., Dolder, C. R., Leckband, S. G., & Jeste, D. V. (2002). Prevalence of and risk factors for medication nonadherence in patients with schizophrenia: a comprehensive review of recent literature. *Journal Of Clinical Psychiatry*, *63*(10), 892–909. 10.4088/jcp.v63n1007 12416599 10.4088/jcp.v63n1007

[CR20] Liao, S., Jang, S., Tharp, J. A., & Lester, N. A. (2023). Relationship between medication adherence for opioid use disorder and health care costs and health care events in a claims dataset. *J Subst Use Addict Treat*, *154*, 209139. 10.1016/j.josat.2023.209139 37574167 10.1016/j.josat.2023.209139

[CR19] Li, J., McCombs, J. S., & Stimmel, G. L. (2002, Sep). Cost of treating bipolar disorder in the California Medicaid (Medi-Cal) program. *Journal Of Affective Disorders*, *71*(1–3), 131–139. ttps://doi.org/10.1016/s0165-0327(01)00394-9 12167509 10.1016/s0165-0327(01)00394-9

[CR21] Magura, S., Rosenblum, A., & Fong, C. (2011). Nov 11). Factors associated with medication adherence among psychiatric outpatients at substance abuse risk. *Open Addict J*, *4*, 58–64. ttps://doi.org/10.2174/1874941001104010058 23264842 10.2174/1874941001104010058 PMC3526017

[CR22] Moran, G., Knudsen, H., & Snyder, C. (2019). Psychosocial supports in medication-assisted treatment: Recent evidence and current practice. *Appendix B Findings and Conclusions* https://aspe.hhs.gov/reports/psychosocial-supports-medication-assisted-treatment-recent-evidence-current-practice-0

[CR23] National Academies of Sciences, Engineering, and Medicine (2025). Enhancing care and services for mental health and substance use disorders: Proceedings of a Workshop—in Brief. T. N. A. Press. https://www.nationalacademies.org/publications/29242

[CR24] National Committee for Quality Assurance (2025). Health plan and employer data information set. antidepressant medication management (AMM) Retrieved May 9 from https://www.ncqa.org/report-cards/health-plans/state-of-health-care-quality-report/antidepressant-medication-management-amm/

[CR25] Novak, P., Feder, K. A., Ali, M. M., & Chen, J. (2019). Behavioral health treatment utilization among individuals with co-occurring opioid use disorder and mental illness: Evidence from a national survey. *Journal Of Substance Abuse Treatment*, *98*, 47–52. 10.1016/j.jsat.2018.12.006 30665603 10.1016/j.jsat.2018.12.006 PMC6350939

[CR26] Pettit Bruns, D., & Kraguljac, N. V. (2023). Co-occurring opioid use disorder and serious mental illness: A selective literature review. *Journal Of Nursing Scholarship*, *55*(3), 646–654. 10.1111/jnu.12879 36734070 10.1111/jnu.12879

[CR27] Pro, G., Neighbors, H. W., Wilkerson, B., & Haynes, T. (2025). Place-based access to integrated mental health services within substance use disorder treatment facilities in the US. *Social Science And Medicine*, *369*, 117843. 10.1016/j.socscimed.2025.117843 39983247 10.1016/j.socscimed.2025.117843

[CR28] Quan, H., Li, B., Couris, C. M., Fushimi, K., Graham, P., Hider, P., Januel, J. M., & Sundararajan, V. (2011). Mar 15). Updating and validating the Charlson comorbidity index and score for risk adjustment in hospital discharge abstracts using data from 6 countries. *American Journal Of Epidemiology*, *173*(6), 676–682. ttps://doi.org/10.1093/aje/kwq433 21330339 10.1093/aje/kwq433

[CR3] Reif, S., Brolin, M. F., Stewart, M. T., Fuchs, T. J., Speaker, E., & Mazel, S. B. (2020). The Washington State hub and spoke model to increase access to medication treatment for opioid use disorders. *Journal of Substance Abuse Treatment*, *108*, 33–39. 10.1016/j.jsat.2019.07.007 10.1016/j.jsat.2019.07.007 PMC6893117 31358328

[CR6] Reif, S., Stewart, M. T., Daily, S. M., Brolin, M. F., Lee, M. T., Panas, L., ... & Wicks, J. J. (2025a). Relationship of hub and treatment characteristics with client outcomes in the initial Washington State hub and spoke cohort. *Journal of Substance Use and Addiction Treatment*. 10.1016/j.josat.2024.209544 10.1016/j.josat.2024.209544 PMC11926888 39437904

[CR5] Reif, S., Stewart, M. T., Daily, S. M., Panas, L., Ritter, G. A., Brolin, M. F., ... & Wicks, J. J. (2025b). Effectiveness of Washington State's hub and spoke model to improve opioid use disorder outcomes. *Health Affairs Scholar*, *3*(8), qxaf157. 10.1093/haschl/qxaf157 10.1093/haschl/qxaf157 PMC12392894 40896384

[CR29] Rezansoff, S. N., Moniruzzaman, A., & Somers, J. M. (2019). Temporal associations between medication adherence for patients with schizophrenia and opioid dependence: A 17-year Canadian Cohort Study. *Schizophrenia Research*, *210*, 157–163. 10.1016/j.schres.2019.05.031 31202570 10.1016/j.schres.2019.05.031

[CR30] Robertson, A. G., Easter, M. M., Lin, H. J., Frisman, L. K., Swanson, J. W., & Swartz, M. S. (2018). Associations between pharmacotherapy for opioid dependence and clinical and criminal justice outcomes among adults with co-occurring serious mental illness. *Journal Of Substance Abuse Treatment*, *86*, 17–25. 10.1016/j.jsat.2017.12.003 29415846 10.1016/j.jsat.2017.12.003 PMC5808599

[CR31] Semahegn, A., Torpey, K., Manu, A., Assefa, N., Tesfaye, G., & Ankomah, A. (2020). Psychotropic medication non-adherence and its associated factors among patients with major psychiatric disorders: a systematic review and meta-analysis. Systematic Review, 9(1), 17. 10.1186/s13643-020-1274-3 10.1186/s13643-020-1274-3 PMC6966860 31948489

[CR32] Smart, R., Kim, J. Y., Kennedy, S., Tang, L., Allen, L., Crane, D., Mack, A., Mohamoud, S., Pauly, N., Perez, R., & Donohue, J. (2023). Association of polysubstance use disorder with treatment quality among Medicaid beneficiaries with opioid use disorder. *Journal Of Substance Abuse Treatment*, *144*, 108921. 10.1016/j.jsat.2022.108921 36327615 10.1016/j.jsat.2022.108921 PMC10664516

[CR33] Snell-Rood, C., Willging, C., Showalter, D., Peters, H., & Pollini, R. A. (2021). System-level factors shaping the implementation of hub and spoke systems to expand MOUD in rural areas. *Subst Abus*, *42*(4), 716–725. ttps://doi.org/10.1080/08897077.2020.1846149 33284083 10.1080/08897077.2020.1846149

[CR4] Stewart, M. T., Daily, S. M., Thomas, C. P., Panas, L., Ritter, G., & Reif, S. (2024). Expanding access to medication treatment for opioid use disorders: Findings from the Washington State hub and spoke effort. 10.1016/j.drugalcdep.2024.111125 10.1016/j.drugalcdep.2024.111125 PMC10922849 38368666

[CR34] Substance Abuse and Mental Health Services Administration (2016). metrics and quality measures for behavioral health clinics: Technical specifications and resource manual. https://www.samhsa.gov/sites/default/files/tech-specs-for-bhcs-vol1.pdf

[CR35] Substance Abuse and Mental Health Services Administration (2024). Key substance use and mental health indicators in the United States: Results from the 2023 National Survey on Drug Use and Health. Retrieved June 17 from https://www.samhsa.gov/data/report/2023-nsduh-annual-national-report.

[CR36] Tarn, D. M., Shih, K. J., Ober, A. J., Hunter, S. B., Watkins, K. E., Martinez, J., Montero, A., McCreary, M., Leamon, I., Sheehe, J., & Bromley, E. (2023). Feb). Perspectives Regarding Medications for Opioid Use Disorder Among Individuals with Mental Illness. *Community Ment Health J*, *59*(2), 345–356. ttps://doi.org/10.1007/s10597-022-01012-x 35906435 10.1007/s10597-022-01012-x PMC9859922

[CR37] U.S. Department of Agriculture; Economic Research Service (2025). Rural-urban commuting area codes. Retrieved April 25 from https://www.ers.usda.gov/data-products/rural-urban-commuting-area-codes

